# The interrelationship between pain, life satisfaction and mental health in adults with traumatic spinal cord injury, in the context of a developing country

**DOI:** 10.1038/s41394-024-00622-9

**Published:** 2024-03-07

**Authors:** Tammy-Lee Williams, Conran Joseph, Lena Nilsson-Wikmar, Joliana Phillips

**Affiliations:** 1 Department of Physiotherapy, University of the Western Cape, Cape Town, 7535 South Africa; 2 Division of Physiotherapy, Stellenbosch University, Stellenbosch, 7602 South Africa; 3 Department of Neurobiology, Care Sciences and Society, Division of Physiotherapy, Karolinska Institutet, 141 52 Huddinge, Solna, Sweden

**Keywords:** Quality of life, Public health

## Abstract

**Study design:**

Cross-sectional, analytical study design using a conveneient sampling strategy.

**Objectives:**

To examine the interrelationship between pain, life satisfaction and indices of negative mental well-being amongst the traumatic spinal cord injury (TSCI) population.

**Setting:**

Western Cape Rehabilitation Center in Cape Town, South Africa.

**Methods:**

Participants (*n* = 70) were adults (mean age of 36.3, *SD* = 9.2) with TSCI. Participants completed the following instruments: 10 satisfaction items from the World Health Organization Quality of Life Brief Version, short forms of the Center for Epidemiological Studies Depression Scale and the trait scale of the State-Trait Anxiety Inventory, a one-item measure of pain intensity taken from the International Spinal Cord Injury Pain Basic Data Set and the interference scale of the Brief Pain Inventory.

**Results:**

Correlational analysis (Pearson r) demonstrated that all the indices of mental well-being as well as the two indices of pain was significantly negatively related to life satisfaction. In addition, life satisfaction mediated the relationship between pain intensity and depression as well as anxiety. Life satisfaction only mediated the relationship between pain interference and depression but not anxiety.

**Conclusions:**

An improvement in life satisfaction may lead to improvements in pain interference, pain intensity as well as psychological distress, amongst persons suffering from TSCI Future research should focus on assessing measures/treatment which may improve life satisfaction in the TSCI population.

## Introduction

In developing countries, traumatic spinal cord injuries (TSCI’s) occur in every 22.55 per million, per year [1]. In the Western Cape region of South Africa specifically, where the study was conducted, as many as 75.6 TSCI’s occur per million, per year [2]. The presence of a TSCI affects individuals’ quality of life to varying degrees [3–5]. Quality of life (QOL) is defined by the World Health Organization (WHO) as “an individual’s perception of their position in life in the context of the culture and value systems in which they live and in relation to their goals, expectations, standards and concerns” [6].

QOL is multi-dimensional in nature and it includes aspects such as physical health (for example, energy, pain and discomfort, etc.), psychological (for example, bodily appearance, positive and negative feelings, etc.), social relationships and environment (for example, home environment, participation in the community etc.) [6]. Embedded in these domains lies satisfaction with various aspects of life, such as satisfaction with life, sleep, ability to perform activities of daily living, etc [6]. Furthermore, life satisfaction has been defined as a cognitive and affective evaluation of the degree of positivity in one’s life, which include aspects such as sleep, work, personal relationships and support from others [6–8]. The vulnerable TSCI group experiences lower life satisfaction compared to the general population [9]. In the developing context, such as South Africa, external factors such as employement, monthly income, living environment and education significantly impact life satisfaction [5, 10–12] as these conditions are already in place, in many instances, prior to the TSCI occurence. Furthermore, in developing countries, persons with SCI demonstrated poorer QOL when compared to developed countries [10, 11]. Persons with higher education, those with employment and those with a longer duration since injury demonstrate a higher quality of life, in developing contexts [3, 5].

In addition, life satisfaction is also impacted by the presence of chronic pain in TSCI population [13, 14]. Chronic pain is defined by the International Association for the study of pain (IASP) as pain persisting for longer than 3 months duration [15], and in the SCI population, chronic pain consists of nociceptive pain and neuropathic pain [16]. Chronic pain is of high prevalence in the SCI population [17]. Although life satisfaction in the developing context has shown to be dependent on these socioeconomic and socioecological factors, studies have demonstrated the protective role of life satisfaction in mediating the negative impact of health aggravators such as pain and thereby maintaing good health [18, 19]. We are therefore of the opinion that more broader societal interventions to improve life satisfaction are needed to mediate / moderate human functioning, including pain experience and mental health status.

There are limited studies investigating life satisfaction and its impact on mental health (anxiety and depression) in the TSCI population, specifically in developing contexts. In various populations however, life satisfaction is associated with psychological distress [20–22].

TSCI is an unexpected negative life event and research has indicated that the emergence of various negative life events has been associated with an increased risk of depression, anxiety and stress [20–22]. The same is true in the TSCI population [23, 24]. In developed countries, factors which affect mental health in the TSCI population include length of time since injury, gender and pain [23–25]. The literature reveals that the greater the duration since the onset of the TSCI results in lower levels of anxiety and depression[23, 25]. The length of time may be associated with acceptance of one’s condition and aid in better coping mechanisms. Earlier studies have proven that acceptance techniques are effective in improving chronic pain and psychological distress, in the SCI population [26, 27]. A more recent study demonstrated the effectiveness of acceptance techniques and cognitive behavioral therapy in persons with chronic pain [28]. As for gender and pain affecting mental health, males and persons suffering from pain have shown greater levels of depression and anxiety in the TSCI population [23, 24, 29, 30]. This may be the result of resilience differences between genders. Two studies found that, in general, males possess higher resilience compared to females [31, 32]. In the TSCI population, Wang and colleagues [23] found that increased resilience was associated with a decrease in psychological distress, in persons suffering from TSCI.

Although internationally QOL and mental health amongst the TSCI population is well investigated, a dearth of research regarding chronic pain, mental health and life satisfaction in the TSCI population is evident in the developing context and in South Africa specifically. This is of importance because of the unique demographic (i.e., mostly males between 16–30 years) and etiological profile (i.e., assualt, especially, firearm related injuries) of persons with TSC in South Africa The current study aimed to examine the interrelationship between life satisfaction, pain and psychological distress in persons with TSCI, in a developing context. We hypothesized that depression, anxiety and pain would be negatively related to life satisfaction.

## Methods

### Design

Cross-sectional, analytical study design, using a convenient sampling strategy to recruit adults with TSCI, living in the City of Cape Town Metropolitan region, from medical databases. Persons were excluded if they were unable to verbalize informed consent and if they were unable to comprehend the questions.

### Participants and procedure

Participants (*n* = 70) were adults who had experienced a traumatic spinal cord injury. To identify potential participants, we studied medical records at the Western Cape Rehabilitation Center in Cape Town, South Africa. We approached healthcare professionals who worked with these patients as well as peer supporters, who provided a supporting role to these patients during their stay in the rehabilitation center, to recruit participants for the study. The instruments were administered by means of telephonic interviews (by the first author and research assistants) and each interview lasted 20–30 min. A summary of the characteristics of the sample is presented in Table 1.

**Table 1:** Description of the sample.

Variable	Category	*N*/Mean	%/SD
Gender	Men	61	87.1
	Women	9	12.9
Employment	Unemployed	66	94.3
	Self-employed	2	2.9
	Employed	2	2.9
Qualifications	Primary school	7	10.0
	Secondary school	62	88.6
	Post-school education	1	1.4
Etiology of injury	Gunshot wound	28	40.0
	Stab wound	12	17.1
	Motor vehicle accident	19	27.1
	Blunt assault	4	5.7
	Pedestrian accident	1	1.4
	Fall from height	6	8.6
Age		35.48	9.34
Time since injury	1–5 years	59	84.3
	6–10 years	11	15.7
Spinal section affected	Cervical	15	21.4
	Thoracic	42	60.0
	Lumbar	5	7.1
	Unknown	8	11.4

*SD* Standard Deviation.

### Measures

Participants responded to items from the following instruments: the life satisfaction items contained in the World Health Organization Quality of Life Brief Version (WHOQoL-BREF) [6], the 10-item version of the Center for Epidemiological Studies Depression Scale (CESD-10) [33], the 5-item version of the trait scale of the State-Trait Anxiety Inventory (STAI-T5) [34], a one-item measure of pain intensity taken from the International Spinal Cord Injury Pain Basic Data Set (ISCIPBDS) [35], and the seven items related to pain interference taken from the Brief Pain Inventory (BPI) [36]. A licensing agreement was entered into with The University of Texas M.D. Anderson Cancer Center to make use of the English and Xhosa versions of the BPI. In addition, participants responded to a brief demographic survey as well as background information on their injuries.

The WHOQOL-BREF is a multidimensional assessment of quality of life. It contains 10 items related to satisfaction with various aspects of life, for example sleep, work, personal relationships and support from others. The WHO-satisfaction items are scored on a 5-point scale with anchors 1 (*very dissatisfied*) and 5 (*very satisfied*). An example item of the WHOQOL-BREF is “*How satisfied are you with your sleep*?”. An analysis of the The WHOQOL-BREF measure in the SCI population shows good internal consistency (Cronbach’s alpha coefficients of 0.74∼0.87, intrainterviewer reliability (intraclass correlation coefficient (ICC) = 0.84∼0.98) in the SCI population [37]. In addition, the reliability of the 10 satisfaction-related items are reported in Table 2 in the results section.

**Table 2:** Intercorrelations between variables, descriptive statistics and reliabilities.

Variable and indices	1	2	3	4	5
1. Pain intensity	–				
2. Pain interference	0.72^***^	–			
3. Life satisfaction	−0.36^**^	−0.45^***^	–		
4. Depression	0.31^*^	0.31^*^	−0.45^***^	–	
5. Anxiety	0.25^*^	0.32^*^	−0.34^**^	0.54^***^	–
Mean	5.55	31.22	30.97	11.30	11.94
SD	3.61	24.33	7.25	5.74	4.23
Minimum	0.00	0.00	16.00	2.00	5.00
Maximum	10.00	70.00	46.00	24.00	20.00
Skewness	−0.39	−0.38	0.20	0.16	0.35
Kurtosis	−1.21	−1.43	−0.97	−0.93	−0.74
α		0.96	0.78	0.69	0.77
ω		0.96	0.77	0.72	0.78

^***^p < 0.001; ^**^p < 0.01; ^*^p < 0.05.

The CESD-10 is a 10-item short version of the Center for Epidemiological Studies Depression scale (CESD-20) [33]. Responses to the 10 items are made on a 4-point scale ranging from 0 (*rarely or none of the time*) to 3 (“*most or all of the time*”). An example item of the CESD-10 is “*I felt that everything I did was an effort*”. The CESD-10 has demonstrated very good validity and reliability when assessed in the TSCI population (*α* = 0.86 and ICC = 0.85) [38].

The STAI-T5 is a 5-item version assessing anxiety [34]. Responses to the 5-items are made on a 4-point scale ranging from 1 (*not at all*) to 4 (*very much so*). An example item of the STAI-T5 is “*I worry too much about something that really doesn’t matter*”. Zsido and colleagues [34] demonstrated that the short form of the STAI-T5 is comparable to the original 20-item version and in this regard, they reported an internal consistency coefficient of 0.82 as well as evidence for the validity of the short form.

We assessed three aspects related to pain that participants were experiencing as a result of their TSCI, namely the quality and nature of worst pain problems and their locations, the intensity of pain, as well as the extent to which the pain interfered with daily activities: including general activity, walking, work, mood, enjoyment of life, relations with others, and sleep. Pain intensity was assessed using one item taken from the ISCIPBDS [35]. This measure collects information on the interference of pain with physical and emotional function and sleep, probable pain diagnosis, location, intensity and duration of pain. The one item assessing intensity of pain was a visual analog scale where participants indicated the intensity of pain experienced on a continuous line between 0 and 10. Pain interference was assessed using the 7-item pain interference scale of the BPI. Responses to the seven items are made on a 11-point scale ranging from 0 (*does not interfere*) to 10 (*completely interferes*). An example item of the BPI-Interference Scale is “*How has pain interfered with your mood in the past week?*”. In the SCI population, the psychometric properties of the BPI has shown excellent reliability and validity [39].

### Statistical analysis

All analyses were conducted using IBM SPSS for Windows version 28 (IBM Corp., Armonk, NY, USA). This included the descriptive statistics (means and standard deviations) and reliabilities of variables (alpha and omega) as well as intercorrelations (Pearson r) between variables. We report both coefficients alpha and omega as coefficient alpha has been found to at times underestimate true reliability [40]. For this purpose, The OMEGA macro for SPSS [40] was used to determine omega. Mediation analyses, with life satisfaction as the mediator, were conducted with the PROCESS macro version 3.5 developed for SPSS by Hayes [41]. Life satisfaction as a mediator is motivated by previous studies that have demonstrated the protective role of life satisfaction in maintaining good physical and psychological health in the presence of health aggravators such as pain etc. [18, 19, 42, 43]. The significance of direct and indirect effects in the mediation model was evaluated using bootstrapped 95% confidence intervals (5000 samples).

We used indices of skewness and kurtosis to determine whether data was normally distributed. Skewness values should optimally be −2 to +2, while kurtosis values should ideally range between −7 to +7 [44]. Skewness values ranged between −0.38 to 0.20 and Kurtosis values ranged between −1.44 to 0.93 which would indicate that data for all the scales was appropriately normally distributed.

## Results

As can be seen in Table 1, the majority of the sample were men (87.1%) who were unemployed (94.3%). The mean age of the sample was 35.48 years (*SD* = 9.34), and the youngest participant was 20 years old, while the oldest was 61 years of age. The majority of the participants had secondary schooling (88.6%). Most of the injuries were caused by gunshot wounds (40.0%), with the second highest being motor vehicle accidents (27.1%). Most of the participants sustained the TSCI within the last three years (60%). The most commonly affected spinal section was the thoracic spine (60%).

Eighty three percent of participants suffered from chronic pain, while 17 reported no pain. The most prevalent types of chronic pain present in the current cohort consisted of below-level neuropathic pain (BL-NEUP) and musculoskeletal nociceptive pain (MNP). Respondents had multiple pain problems and 47.1% indicated BL-NEUP as their first worst pain, 31.4% reported it as their second worst pain and 8.6% indicated BL-NEUP as their third worst pain. Similarly, 31.4% indicated MNP as their first worst pain, 4.3% reported it as their second worst pain and 7.1% indicated that MNP was their third worst pain. For those reporting their ‘first worst pain problem’, 45 persons reported using treatment for their pain and 21 persons reported using no treatment. For those with a ‘second worst pain problem’, 23 persons reported using treatment for their pain and 30 persons reported using no treatment. Lastly, for those with a ‘second worst pain problem’, 11 persons reported using treatment for their pain and 34 persons reported using no treatment.

The regions of the body most commonly affected by chronic pain comprised of the lower limb/bilateral lower limbs, lower back and shoulder.

The intercorrelations between variables, descriptive statistics and estimates of internal consistency are reported in Table 2.

Table 2 reflects that there was a significant moderate negative relationship between life satisfaction and pain interference (*r* = −0.45, *p* < 0.001, medium effect) as well as pain intensity (*r* = −0.36, *p* = 0.003, medium effect). There was also a significant positive relationship between pain interference and pain intensity, on the one hand, and depression (pain intensity: *r* = 0.31, *p* = 0.012, moderate effect; pain interference: *r* = 0.31, *p* = 0.012, moderate effect) and anxiety (pain intensity: *r* = 0.25, *p* = 0.042, small effect; pain interference: *r* = 0.32, *p* = 0.010, moderate effect), on the other hand. These obtained relationships would indicate that higher levels of pain intensity and pain interference were associated with lower levels of life satisfaction, and higher levels of depression and anxiety. There was also a significant moderate negative relationship between life satisfaction and depression (*r* = −0.45, *p* < 0.001) as well as anxiety (*r* = −0.34, *p* = 0.005). Thus, higher levels of life satisfaction was associated with lower levels of depression and anxiety.

Except for coefficient alpha in the case of the CESD-10, all the estimates of internal consistence exceeded the conventional cutoff (≥0.70) for acceptable reliability (α and ω = 0.77 to 0.96). However, in the case of the CESD-10 while coefficient alpha was just below 0.70, coefficient omega was 0.72.

**Fig. 1:**
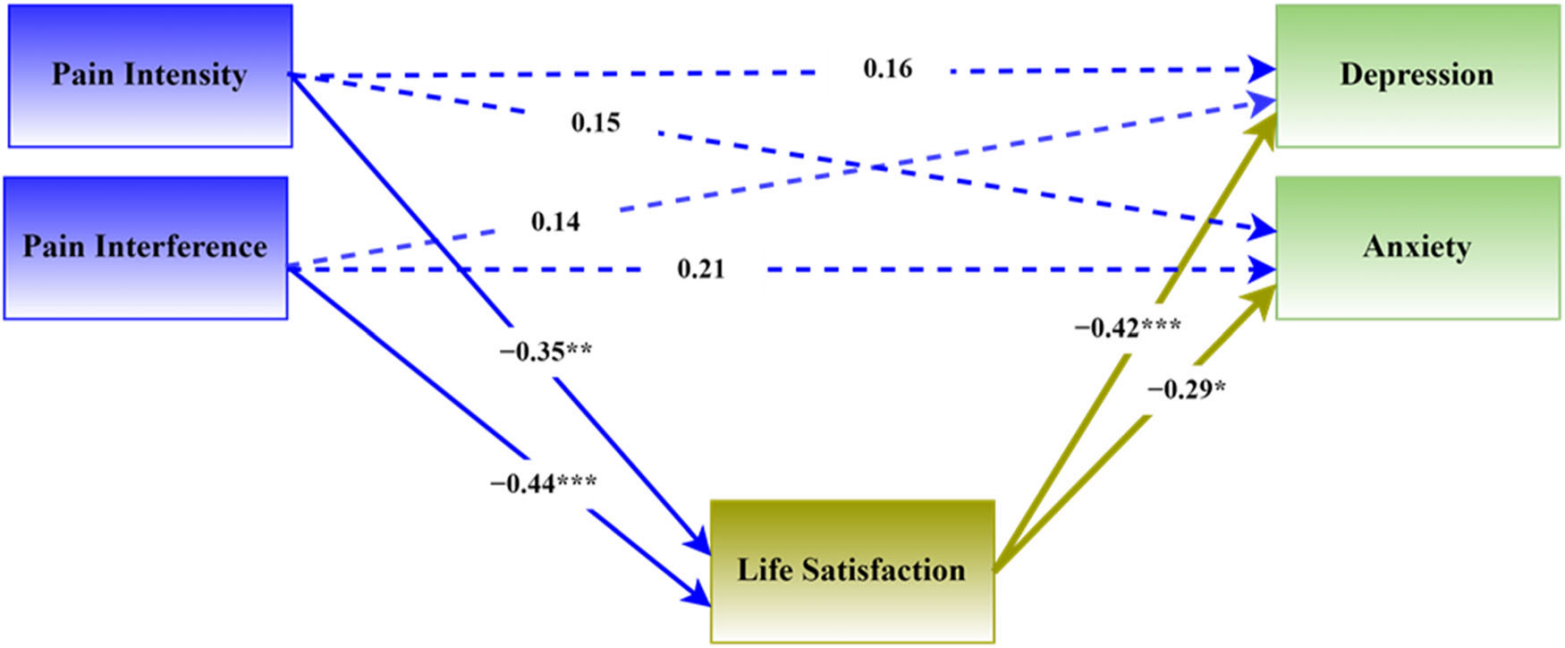
Conceptual model of the mediating role of life satisfaction. *Note*: Regression coefficients are standardized. ***p < 0.001, **p < 0.01, *p < 0.05. Dotted lines show non-significant associations in the presence of the mediator.

The conceptual model of the mediating role of life satisfaction is shown in Fig. 1. The standardized coefficients in the model was obtained with the PROCESS macro.

The direct and indirect effects of pain interference and pain intensity on depression and anxiety are reported in Table 3.

**Table 3:** Direct and indirect effects of pain intensity and interference.

Effect	B	SE	95% CI	β	*p*-value
Direct Effects					
Pain intensity → depression	0.26	0.19	[−0.11, 0.63]	0.16	0.166
Pain interference → depression	0.03	0.03	[−0.03, 0.09]	0.14	0.269
Pain intensity → anxiety	0.18	0.15	[−0.12, 0.48]	0.15	0.232
Pain interference → anxiety	0.04	0.02	[−0.01, 0.08]	0.21	0.118
Life satisfaction → depression	−0.33	0.09	[−0.51, −0.15]	−0.42	< 0.001
Life satisfaction → anxiety	−0.17	0.07	[−0.32, −0.02]	−0.29	0.022
Indirect effects					
Pain intensity → Life satisfaction→ depression	0.23	0.10	[0.07, 0.46]	0.15	
Pain interference → Life satisfaction→ depression	0.04	0.02	[0.01, 0.08]	0.17	
Pain intensity → Life satisfaction→ anxiety	0.12	0.08	[0.01,0.30]	0.10	
Pain interference → Life satisfaction→ anxiety	0.02	0.01	[−0.00, 0.05]	0.11	

B = unstandardized coefficient, β = Standardized coefficient.

Table 3 reflects the significant mediating role of life satisfaction. In particular, it shows that life satisfaction mediated the relationships between pain intensity and depression (β = 0.15, 95% CI 0.07 to 0.46) as well as anxiety (β = 0.10, 95% CI 0.01 to 0.30). In addition, life satisfaction mediated the relationship between pain interference and depression (β = 0.17, 95% CI 0.01 to 0.08), but not between pain interference and anxiety (β = 0.11, 95% CI − 0.00 to 0.05).

Table 3 also shows the significant direct associations between life satisfaction and depression (β = −0.42, *p* < 0.001) as well as anxiety (β = −0.29, *p* < 0.001).

In the absence of the mediator, pain intensity and pain interference were significantly associated with depression and anxiety (see Table 1). However, in the mediation model these associations were non-significant and this indicates that life satisfaction fully mediated the impact of pain on indices of psychological well-being.

## Discussion

The current study examined the interrelationship between the experience of pain in adults with traumatic spinal cord injuries, life satisfaction and negative indices of psychological well-being in a developing context, specifically, South Africa. There were several significant findings.

First, our study found that the experience of pain (pain interference and pain intensity) was significantly associated with higher levels of depression and anxiety and lower levels of life satisfaction. This finding is similar to a study conducted in the developing country of Kenya, where pain was significantly related to depression, with increased pain relating to higher depression [45]. In the developed context, these findings are supported by studies which found pain severity positively correlated with moderate and severe depression [24]. In addition, persons with TSCI experiencing pain reported more anxiety and depression compared to those without pain [30]. Chronic pain as well as the degree of disability associated with the TSCI affects life satisfaction and a recent study in New Zealand found that those with a higher degree of TSCI disability reported lower life satisfaction [13]. Thus, the degree of disability should be considered when managing pain in order to improve life satisfaction. Support from others have also been shown to impact the degree of life satisfaction in the presence of chronic pain [14].

Second, our study found that life satisfaction was directly associated with depression and anxiety. This would indicate that being satisfied with life, in itself, is beneficial for psychological well-being. To the best of our knowledge, there is limited research directly investigating the association between life satisfaction, anxiety and depression, in the TSCI population. One study by Parker et al. [46] found that anxiety, amongst persons suffering from SCI, was associated with lower life satisfaction, although not significantly. Depression on the other hand was significantly associated with poorer life satisfaction [46]. The current study adds new knowledge to substantiate the importance of life satisfaction as a construct to improve psychological wellbeing in the TSCI population. The existing research reveals that there is considerable lower life satisfaction in people with SCI [9, 47]. In a recent comparative study, persons with SCI living in lower-income countries demonstrated poorer life satisfaction compared to those living in higher-income countries [12]. Higher-income countries may provide more employment opportunities, support and access to healthcare which may impact life satisfaction.

Third, our study found that life satisfaction fully mediated the relationship between pain intensity and depression as well as anxiety. However, it only mediated the relationship between pain interference and depression but not pain interference and anxiety. This would indicate that being satisfied with life can serve as a protective factor in terms of the impact of pain on psychological wellbeing. Previous literature has shown that good physical and psychological health is associated with higher life satisfaction in persons with and without medical condition [18, 19]. In addition, life satisfaction plays a protective role in maintaining good health by mediating the impact of negative thoughts related to aggravators such as pain, ill-health etc. This maintenance of health may be represented by the avoidance or decrease in distructive health behaviors such as smoking, drinking alcohol, inactivity etc. [18]

The findings obtained in our developing context, to a certain extent, replicates the findings of studies conducted in other developed contexts.

Cognitive behavioral therapy (CBT), such as mindfulness therapy as well as exposure and acceptance therapy, has been shown as effective measures used to improve life satisfaction, pain disability and negative affect in persons suffering from chronic pain [28], however, further research is required in the field of TSCI. In addition, although life satisfaction mediates the relationship between pain and psychological distress, further research should investigate the longitudinal evolution of these factors to determine which construct increases the probability of the other and thereby allowing for early targeted treatment.

The study has several limitations. The research design (cross-sectional) limits inferences regarding causality. Access to participants were limited due to the protection of personal information act, resulting in a smaller than expected sample size. Lastly, the study used self-reported data which could lead to social desirability bias. Thus, future studies should strengthen these results by conducting a longitudinal study in order to assess the relationship between chronic pain, life satisfaction and indices of mental health over time and in a bigger sample size.

## Conclusions

Pain interference and pain intensity was significantly associated with higher levels of depression and anxiety and lower levels of life satisfaction, in this developing context. However, the association between life satisfaction, depression and anxiety indicate that being satisfied with life is beneficial for psychological well-being. In addition, being satisfied with life can serve as a protective factor in terms of the impact of pain on psychological wellbeing.

An improvement in life satisfaction may lead to improvements in pain interference, pain intensity as well as psychological distress, amongst persons suffering from TSCI. CBT has been shown to result in improvements in life satisfaction in persons suffering from chronic pain, however, further research is required in the TSCI population.

Future research should focus on assessing measures/therapy which may improve life satisfaction in the TSCI population. In addition, further research should focus on assessing pain, psychological distress and life satisfaction longitudinally in order to determine the longitudinal impact of each construct and thereby enable early targeted treatment.

## Data Availability

The raw data supporting the conclusions of this article will be made available by the corresponding author, upon appropriate request.
